# Needed Relapse-Prevention Research on Novel Framework (ASPIRE Model) for Substance Use Disorders Treatment

**DOI:** 10.3389/fpsyt.2015.00037

**Published:** 2015-03-06

**Authors:** Udi E. Ghitza

**Affiliations:** ^1^Center for the Clinical Trials Network, National Institute on Drug Abuse, National Institutes of Health, Bethesda, MD, USA

**Keywords:** addictive disorders, drug abuse, substance abuse, addiction, addiction treatment, drug abuse treatment, personalized medicine, precision medicine

## Introduction

According to a recent United States (U.S.) national survey on drug use and health, substance use disorders (SUDs) characterized by hazardous use of alcohol, non-prescribed drugs, and illicit drugs are common. In 2013, 22.7 million persons (8.6% of persons aged 12 years or older) needed treatment for an illicit drug or alcohol-use problem (1). Drug use disorders produce a wide variety of medical problems and are important contributors to years of life lost due to disability and preventable death (2). Yet, screening, intervening, and treating SUDs have not been embraced within general medical settings (3). In fact, even though untreated SUDs place individuals at substantially greater risk for a wide range of diseases and are a pervasive public health burden, only approximately one-tenth of Americans aged 12 years or older with SUDs received treatment in 2013 (1). A main obstacle to patient engagement in and initiation of SUDs treatment is lack of motivation to seek out follow-up care. Motivation to do so may be compromised by a lack of a personalized-medicine approach, in which patients are frequently only offered a single or narrow set of care options, which do not align with their individual needs. A gap exists in research in medical settings regarding how to effectively implement shared-decision making between patients and their providers, concerning how to tailor evidence-based care options to personalized needs and preferences of clients (4–6).

Converging research over the past 30 years indicates that core features of SUDs comprise of a spectrum of neurobiological components, which in 2014, I distilled into a shared-decision-making and personalized/precision medicine framework for SUD treatment, the ASPIRE model (7). This integrative model stresses the need for translational research on how to most effectively optimize implementation of patient-centered care using this model in general medical settings and specialty SUD-treatment programs, in a manner whereby patients have an active role as partners in deciding with their providers on treatment strategies for evidence-based care tailored to their risk categories and values (7). This precision medicine approach should be customized to genetic, psychological, and physiological profiles and environmental events that patients experience as presenting an obstacle to their overall wellness and recovery (7). Accordingly, treatment and follow-up care plans should be matched with individual disease characteristics, which may enhance motivation of patients to cease hazardous substance use and associated health risk behaviors (7). Thus, the ASPIRE model uses as its foundational principle shared-decision-making between patients and healthcare providers to customize personalized medical care to particular neuroscience-research-grounded profiles, psychiatric comorbidities, readiness to change, risk categories, and problems that individual patients report as most distressing to their daily lives (7). Recent advances in genomic and health information technologies [for instance, meaningful use of electronic health record systems (EHRs), telemedicine, mobile-health technologies, EHRs-linked patient registries, EHRs-linked genomic data and lifestyle information, etc.] and bioinformatics methods for analyzing EHRs-linked biomedical big-data repositories should be leveraged to accelerate rigorous big-data science research on how to effectively implement such a personalized-medicine framework in general medical settings.

In circumstances where individual patients are cognitively impaired and have limited insight, such a shared-decision-making approach to SUD treatment may actively involve patients’ caregivers or other family members, if patients consent to their involvement in their medical care (4–7). In such cases, family members may be recruited to help ensure patients follow through and adhere to their treatment plan mutually agreed upon with their medical provider. Systematic rigorous research is needed using a shared-decision-making approach to find more effective ways of reducing the burden of SUD and enhancing quality of lives not only for individual patients but also for their family members affected by an individual’s SUD.

## Evidence Gaps and Research Areas Concerning ASPIRE Framework for Patient-Centered Treatment of SUDs

For patients at the severe end of the SUDs spectrum, below I illuminate needed relapse-prevention research testing combined evidence-based cognitive-behavioral therapy and pharmacological-intervention adjuncts targeting prominent components in the ASPIRE framework. “*A*” in this framework denotes *a*nhedonia/reward-deficit and “*S*” a *s*tressful state, referring to a sensitized brain anhedonia and stress system following repeated heavy drug use and during drug withdrawal (8). This sensitized brain system produces deficits in responses of the brain to rewarding stimuli and produces a negative emotional state, which drives continued drug-taking behavior (8). “*P*” denotes a *p*athological lack of self-control to cut down drug use despite negative implications, which involves impairments in prefrontal–cortical cognitive regulation of inhibitory behaviors to reduce drug taking. Relevant to these phenotypes, recent converging clinical-research findings suggest an overlap among neurobiological events mediating drug craving and relapse triggered by stress and drug-predictive environmental cues (9–13). Across various SUDs (e.g., cocaine, opioid, and tobacco use disorders), these findings indicate adrenergic alpha-2 receptor agonists (e.g., guanfacine, clonidine, lofexidine) may have promise for facilitating relapse prevention by decoupling brain responses to stress and drug-predictive cues from drug craving (9–12). Further, guanfacine is approved by the U.S. Food and Drug Administration (FDA) for treating attention deficit hyperactivity disorder (ADHD) in youth. It has been shown to enhance cognitive processes involved in response inhibition and executive functioning, such as attention shifting, thereby facilitating cortical regulatory control over inflexible behaviors associated with vulnerability to drug seeking and taking in individuals with SUDs (11, 14).

Based upon the above promising proof-of-concept clinical studies, double-blind placebo-controlled randomized clinical trials (RCTs) are needed in treatment-seeking populations to test whether guanfacine or other adrenergic alpha-2 receptor agonists may be effective as pharmacotherapy adjuncts to augment efficacy of currently available evidence-based treatment options, to enhance relapse prevention in recently abstinent patients.

For instance, in the case of opiate-use disorders, there are effective FDA-approved medication-assisted treatment (MAT) options (such as buprenorphine in medical settings). However, even with these treatment options, opiate-use relapse rates remain high. Thus, comparative effectiveness research is needed in general medical settings to determine whether adrenergic alpha-2 receptor agonist adjuncts – thought to work by different mechanisms of action to reduce opiate use – enhance the effectiveness of buprenorphine in relapse prevention, compared with buprenorphine alone.

It would be helpful for such RCTs to follow the FDA guidance on enrichment strategies for clinical trials to support approval of human drugs (15). For example, patients at study intake could be queried on whether they experience frequent psychological distress from stress or restlessness/impulsivity. This is because adrenergic alpha-2 receptor agonists are postulated to be beneficial in relapse prevention by decoupling sensitized brain responses to stress from drug craving, reducing impulsivity, and enhancing cognitive processes important for attention shifting (11–14). If patients do report experiencing such psychological symptoms, the likelihood of detecting an efficacy signal may be improved by employing a personalized-medicine approach in which these patient subgroups would be included in these relapse-prevention trials, since they are likely to benefit most from these agents.

Furthermore, in the context of precision medicine, adaptive designs research is needed to develop and test outpatient treatment algorithms and actionable clinical decision support for different patient subgroups with opiate-use disorders, with or without chronic pain, which incorporate shared-decision making practices in improving their health outcomes and reducing the risk of death from overdose and other opiate-use related negative consequences.

“*I*” and “*R*” in the ASPIRE model denote insomnia and restlessness, common SUDs withdrawal symptoms following repeated heavy drug use that patients find distressing, increasing likelihood of relapse following abstinence (16–20). Gabapentin is a widely prescribed FDA-approved anticonvulsant medication, which reduces alpha-2d-1 membrane trafficking and calcium currents at voltage-gated calcium channels in regions involved in the brain’s sensitized responses to stress following repeated heavy drug use and withdrawal (21, 22). Gabapentin has also been frequently used off-label to reduce sleep disturbances and restlessness. Recent clinical research suggests gabapentin may have promise in reducing alcohol and cannabis use and craving in alcohol- and cannabis-dependent individuals, as well as sleep and mood disturbances (21, 22). Randomized controlled trials are needed to test gabapentin’s effectiveness in facilitating relapse prevention in recently abstinent cannabis-use and/or alcohol-use disorder treatment seekers, employing the FDA guidance on enrichment strategies for clinical trials to support approval of human drugs (15). If patients report experiencing sleep disturbances or restlessness impairing their daily lives, they would be considered for such a RCT, which would test effectiveness of gabapentin combined with suitable evidence-based cognitive-behavioral therapies to target these disturbances, enhance duration of abstinence, and prevent relapse.

In the ASPIRE model, “*E*” denotes excessive preoccupation with seeking drug reinforcement, compared with natural reinforcers, after transition from volitional to compulsive drug use, especially following drug craving when an individual with a SUD comes in contact with a drug-associated environment. Convergent preclinical research, using the reinstatement model of drug relapse, suggests glutamate signaling dysfunction in the mesocorticolimbic dopamine system plays a key role in this addictive process across multiple substances of abuse, including alcohol, cannabis, cocaine, nicotine, and opiates (23). *N*-acetylcysteine is an N-acetyl pro-drug of the naturally occurring amino acid cysteine and is FDA-approved as a mucolytic agent for bronchopulmonary disorders (24). Preclinical research indicates that *N*-acetylcysteine works to reduce drug-environment-triggered reinstatement of drug seeking for various drugs of abuse (23). It may do so by normalizing imbalances in glutamate signaling and associated pathophysiology in neuroplasticity in the mesocorticolimbic dopamine system, in part by reversing down-regulation of glial–glutamate transporter-1 (GLT-1) and metabotropic glutamate receptor 2/3 (mGluR2/3) function (25, 26).

The National Drug Abuse Treatment Clinical Trials Network of the National Institute on Drug Abuse (NIDA CTN) is currently implementing a nationwide placebo-controlled RCT evaluating efficacy of *N*-acetylcysteine versus placebo, added to contingency management, for cannabis cessation in adults ages 18–50 years old. The basis for this trial is positive findings from a RCT in cannabis-dependent adolescents, which found more than twice greater odds of cannabis abstinence during treatment with *N*-acetylcysteine compared with placebo (27, 28). Since basic research suggests that the mechanism of action by which *N*-acetylcysteine works may suit it as a relapse-prevention agent, RCTs are also needed to test its efficacy as a relapse-prevention adjunct to evidence-based treatment options in recently abstinent treatment seekers. Further, pharmacological adjuncts need to be tested, which operate by similar mechanisms to reverse brain-reward-system down-regulation of GLT-1 and mGluR2/3 function and associated glutamate signaling imbalances, but which have greater potency, bioavailability, and membrane permeability than *N*-acetylcysteine. For instance, *N*-acetylcysteine amide (NACA) is a more potent derivative with greater bioavailability and membrane permeability, which may translate into improved clinical research use as a relapse-prevention candidate (23, 29).

## Conclusion

Relapse-prevention research is needed in the above areas using a personalized/precision medicine approach in which medical decisions regarding treatment options are tailored to patient subgroups’ disease profiles and risk categories. The ASPIRE model for SUDs treatment takes into account needs for instituting a shared-decision approach over the course of patient-centered care, addressing preferences and values of patients and their families (7). This framework calls for rigorous systematic research on effectiveness of personalizing treatment to those phenotypes that patients report as presenting distressing problems to their daily lives, driving their continued drug-taking behaviors, and increasing likelihood of drug relapse (7). The FDA guidance on enrichment strategies for clinical trials to support approval of human drugs (15) should be used to guide the design of randomized controlled trials in a manner matching interventions and their mechanisms of action to personalized needs of patients. It would be useful for enrichment strategies to be implemented in a manner whereby only subgroups most likely to benefit from interventions’ postulated mechanism-of-action are included at study intake, prior to randomization. The likelihood of detecting an efficacy signal may be improved following such an approach. Clinical research programs, such as NIDA’s National Drug Abuse Treatment Clinical Trials Network (CTN), can serve an important role in furthering such studies. Existing health information system infrastructures in practice-based research networks and recent advances in bioinformatics methods for analyzing biomedical big-data repositories of large patient cohorts should be leveraged to help accelerate this line of personalized-medicine research. This, in turn, could lead to more cost-efficient enrollment of patients into trials, increased recruitment of providers to deliver interventions within studies, and more efficient utilization of EHRs, EHRs-linked genetic information, mobile-health and telemedicine technologies, and EHRs-linked patient registries (3, 7, 30).

## Conflict of Interest Statement

The author declares that the research was conducted in the absence of any commercial or financial relationships that could be construed as a potential conflict of interest.
